# On Stramonium

**Published:** 1811-07

**Authors:** 


### *For the Medical and Physical Journal.*

### *On Stramonium.*

(Concluded from P. 507. Vol. 25.)

> " So well the fashionable med'cine thrives,

> That now 'tis practis'd ev'n by country wives;

> Pois'ning without regard of fame or fear."

HAVING recorded the effects of the internal administration of Stramonium upon the human system in health and in disease, I shall conclude with some observations upon the practice of Smoking the herb, of late recommended for the cure of Asthma, and other pulmonic affections.

Since the first part of this paper was written, Dr. Sims, whose attention to botanical research, amidst the engagements of extensive practice, is highly creditable, has, in a letter to the Editor of the Monthly Magazine, given the following account of the introduction of Smoking Stramonium in this country,

" Some time in the year 1802 I received from general Gent a remedy that he had not long before brought from Madras, which, the General informed me, was used there as a specific for relieving the paroxysms of asthma, and that it was prepared from the roots of the wild purple-flowered thorn-apple, *datura ferox*. The roots had been cut into slips as soon as gathered, dried in the shade, and then beat into fibres resembling coarse hemp. The mode of using it was by smoking it in a pipe at the time of the paroxysm, either by itself or mixed with tobacco, according as the patients were previously addicted to smoking or not. General Gent procured this remedy from Dr. Anderson, physician general at Madras, who both recommended it, and, I believe, used it himself.

" I happened at this time," continues Dr. Sims, " to be attending the daughter of an eminent physician, labouring under phthisis pulmonalis, combined with asthma, as appeared to me from the frequent paroxysms of difficulty of breathing, not usual in pure phthisis, at least in so early a stage of the disorder. With a view of alleviating these distressing paroxysms, I recommended a trial of this remedy, which to me was at that time perfectly new. The relief obtained was far beyond expectation; and, although gradually sinking sinking under an incurable disease, this amiable lady continued to experience great satisfaction from its use, almost to the fatal termination."

Soon afterwards Dr. Sims recommended this remedy to Mr. Toulmin of Hackney, who had for several years suffered frequent paroxysms of asthma. He received much benefit from its use, and having soon exhausted the original stock given him by Dr. Sims; at the doctor's suggestion, he had recourse to the common thorn-apple, *(datura Stramonium.)* From this he experienced nearly the same relief as from the East Indian plant. Mr. Toulmin communicated the knowledge of the remedy to the correspondent in the Monthly Magazine with the signature of Verax*.

Dr. Sims concludes his letter by observing that he has two purposes to answer by it. "In the first place it will serve to point out the history of the introduction of a remedy which promises to become an important addition to the Materia Medica," and it will prove "that the original remedy, as used in the East Indies, is not exactly the same as what is used here. It is, indeed, highly probable that both species have nearly similar virtues, but the one may, perhaps, be more efficacious than the other."

This account of the remedy is certainly favourable, and from Dr. Sims's authority, many practitioners will, probably, be induced to give it a trial. We must not forget, however, that the doctor has only cited two cases in which benefit has been derived from its use, evidence is still wanting to enable us to decide upon the merits of the plant, to determine in what cases its use may be profitable, and in what cases it may be hurtful.

The following lamentable history of the fate of a gentleman†, who was instrumental in introducing the practice of smoking Stramonium, is probably not known by many who are industriously encouraging the use of this composing smoke.

Dr. Gibbes of Bath being called in to attend this gentleman, found him sitting up with his head reclined on a sofa in a state of stupor. His recollection was impaired, and he seemed stunned and comatose. His pulse was scarcely to be

---

* This writer has since avowed himself to be John Sills, esq. Guildford Street.

† The particulars of this case were communicated to Dr. Bree by Dr. Gibbes. Delicacy to the parties alone prevents the patient's name from appearing, but the authenticity of the account may be relied upon; and the opinion of Dr. G. that smoking Stramonium was the cause of death is confirmed by the testimony of Dr. Parry.

felt, and there was no great force in the carotid artery. Upon inquiring into his case, Dr. G. found that on the preceding evening the gentleman had been very much affected with shortness of breath, and that he had smoked Stramonium, the effect of which was, that his breath became perfectly easy, but at the same time he had shewn symptoms of stupor. He went to bed, passed a quiet night, and arose the next morning in the heavy comatose state in which Dr. Gibbes found him on his arrival at the house. A large blister was applied to the back, purgative medicines were administered; and the urine being deficient in quantity, a draught with squills and camphor mixture was ordered.

On the second day the blister had acted, the bowels had been open, and the urine in proper quantity and of a clear good colour. The mental faculties were somewhat recovered. The treatment was continued, and he was directed to take nourishment, having previously reduced himself very much with the view of alleviating the distress in his breathing. On the third day his recollection was much better, his pulse perceptible, and some return of shortness of breath had occurred. From this symptom Dr. Gibbes drew a favourable opinion, as he observed that as this came on, the mental faculties seemed to improve; nor could he perceive any fulness of vessels to denote apoplexy; nor any evidence of effusion since the urine had returned to its natural quantity and colour. On the fourth day he was so much recovered that he was preparing to go to Clifton; but suddenly expired after dinner, either whilst sleeping, or immediately upon awaking; Dr. Parry, who was then sent for, as being nearer than the attending physician, finding him dead when he arrived.

The patient was of a full habit, and about twelve months previous to this attack, had been affected with a cough and wheezing, from which he recovered under the care of Dr. Gibbes with ordinary expectorants. At the commencement of his last illness he smoked largely of Stramonium, three or four times, without consulting any one; when the serious symptoms above related supervened, and Dr. Gibbes was called in.

My own experience of the effects of this remedy has not been encouraging; I have given it in asthma, in chronic cough attended with severe dyspnœa, and copious expectoration; and in phthisis pulmonalis. Some of the patients, whose complaints were far advanced, died, as they must have done had they not taken the medicine, which, however, I did not think hastened the catastrophe. In two or three instances of dyspnœa, slight and temporary benefit was obtained, but in no one instance was the disorder materially altered in character, and the patients soon abandoned a remedy from which which they derived no relief. Desirous that the properties of Stramonium should be properly estimated, and to prevent any suspicion that my judgment might be influenced by my own want of success, I applied for information to some of my medical friends, whom I knew were especially conversant in the disorders in which this remedy has been said to prove beneficial. Dr. Bree, with great liberality and promptitude, has allowed me to publish the following letter containing the result of his observation on the effects of smoking Stramonium in asthmatical complaints.

DEAR SIR, *May* 10, 1811.

I very willingly comply with your request that I should report the result of my observations on the efficacy or influence of Stramonium in cases of asthma that have fallen under my view.

In certain cases I tried the extract of Stramonium many years ago, but I was not encouraged by my experience at that time to pursue the practice of giving it in general cases of asthma.

In the last year the public were informed, by writers in journals and newspapers, that the smoking of this herb produced ease, and even effected cures in convulsive asthma. The authorities for such success were of a mixed character, some of them being satisfactory, as far as they reported benefit in the fits of asthma; but others, more numerous, were very suspicious, as they were not sanctioned by names, and most of them asserted *cures* after the use of the remedy for a very short time in this disease, of which the access of the paroxysm is both periodical and uncertain.

The evidence of advantage from smoking Stramonium had a doubtful aspect to a considerate physician, and this character was not rendered more clear by the appearance of a "Familiar Treatise" on the subject, pretended to be published by Mr. Surgeon Fisher. Much of the matter in that treatise I knew to be wholly false, whilst the chief object of it was clearly displayed by the recommendation of Stramonium in a *secret* composition, after the manner of other empirical nostrums.

Mr. Toulmin of Hackney gave the only testimony that deserved attention respecting the use of Stramonium in asthma; but this gentleman, with the power of confuting the pretensions of others, did not offer himself to the public notice; and the same reserve, which distinguishes the professional man of science, seems to have restrained him from publishing hasty conclusions from particular facts, that are too often generalized and made subservient to unworthy purposes. I was

 acquainted acquainted, in a private manner, with Mr. Toulmin's use of Stramonium by inhaling it, and the success which some sufferers had experienced in fits of asthma from following his practice, induced me to mention it as a possible means of obtaining relief, when other antispasmodics had been tried without effect.

From the beginning of the present year I have been more attentive to the effects of this practice.

The number of cases which I have had occasion to examine between that period, and the end of April, was 82. The patients were all disturbed in their breathing, but only a proportion of them was truly affected with convulsive asthma. To the whole number the remedy had been either useless, as regarded the removal of the disease, or it had produced injurious or fatal effects. If any signal advantage from the use of Stramonium had been experienced, I should probably not have been consulted, and my report is therefore not intended to deny the success that may be asserted to have taken place in cases I have not seen. You will consider it as a faithful report respecting 82 cases of patients who had smoked this herb under various diseases, which were supposed to be asthmatic.

Those who had smoked Stramonium without any permanent good effect amounted to 58. The remaining 24 had all of them been more or less injured, and some of them detroyed by the practice. I shall only mention cases which were brought to a certain state, admitting of safe inferences as to their further progress, at the end of April. They had been all of them observed with sufficient attention to enable me to ascertain, how far Stramonium was capable of mitigating or removing asthma.

The first list of 58 included 11 cases of obstructed liver; these patients had lost their time in relying upon Stramonium; but I do not place this inconvenience amongst the injuries derived from the practice of smoking this herb, because the constitutions of the patients were yet so vigorous, as to be capable of bearing the necessary evacuations. All of them had constant dyspnœa, and most of them had experienced paroxysms of convulsive breathing at intervals. Three were in an advanced state of the disease, having hard bellies, and swelled legs. Seven gradually recovered by the treatment that was applied for the removal of congestion in the liver, their dyspnœa leaving them, as the disease of this organ gave way. Thesse 11 cases shew the effect of advice which people, ignorant of the distinction of diseases, give with confidence to their friends without any authority excepting that of the advertisements of their newspapers.

The

The remainder of the 58 patients had the usual signs of the asthmatic constitution. They were generally satisfied with a plan, less miraculous in the promise of immediate cure, but more likely to restore tone to their habits, and with the assurance that relieving the convulsive paroxysm of asthma is not removing the disease. I had seen many of them before, and some of these did not refrain from complaining of the assent I had given in the winter to their trials of Stramonium in the difficulty they experienced of appeasing the fit.

The 24 patients who have been stated to have suffered injury from the smoking of Stramonium were all disordered in their breathing, and their dyspnœa, at intervals, assumed the form of convulsive asthma.

Of this number I shall first mention seven patients, whose symptoms indicated phthisis, and whose lungs were weak, and had been long subject to inflammatory attacks on changes of weather, and the taking of colds. The oldest of these was 35 years of age. Their habits were thin, irritable, and weak; and the pulses of all of them in their best state of a dangerous quickness. In their former attacks of difficult respiration, small bleedings, with blisters and febrifuge draughts, that gently promoted expectoration, had always afforded relief. They came under my care in March and April, and all without exception, attributed the aggravation of their complaints to the smoking of Stramonium, or to the use internally of an oxymel of Stramonium. Some of these patients were relieved by the same means, as had been before repeatedly applied to their cases, but three of them spat blood, after violent heat and stricture under the sternum had continued for many days. They now expectorate pus, and are greatly wasted with hectic fever and night sweats, and give no prospect of a fortunate result from any mode of treatment.

Three persons, who had passed the meridian of life, and had suffered asthmatic affections, and coughs, for many years, with great debility and emaciation of the system, experienced paralytic tremblings from smoking Stramonium. Their original complaints were also generally aggravated, excepting their cough, which subsided as their weakness increased. The pulse in each of these patients was so lowered, that it became difficult to feel the beating of the artery. After abandoning their practice of smoking, which two of them had pursued every evening for two weeks, and one twice a day, for ten days; they took strengthening draughts with gentle expectorants. The cough then returned to each patient, and they all recovered their former degree of health.

A lady advanced in life, of weak constitution, and particularly feeble nerves, had been long subject to coughs and asthma. She

She had smoked the Stramonium a few times only, and it affected her head with pain and confusion, and her stomach with sickness. She was next seized with an epileptic fit, the first she had ever experienced. This attack was followed by three more fits of the same kind, at intervals of a few hours, and she became nearly insensible. The cough left her, the pulse became scarcely perceptible, and her mind was no longer capable of any exertion. She was not wholly unconscious of her state, but stupor and somnolency overpowered the little energy she possessed, and her stools and urine passed involuntarily. At first it appeared necessary to remove congestion from the head by cupping, leeches, and blisters. Strengthening medicines were then employed in consultation with Dr. Latham. The patient slowly recovered from this critical state, and attributed her epileptic fits, and preceding confusion of head, to the smoking of Stramonium.

Four persons, all of full habits, and two of them, strictly speaking, apoplectic in their forms, smoked Stramonium for the cure of dyspnœa, which they called asthma. After some days experience of this practice, one of them was still capable of coughing, but with so much pain of his head, as to indicate immediate danger. He was sixty years of age, and the other three were more than fifty. They so convincingly required depletion, that I was surprised it had not been advised by the most superficial of their friends Evacuations by bleeding and purging, removed the difficulty of breathing, and probably preserved the lives of more than one.

The smoking of Stramonium has been practised by many female patients. I saw two patients, of the ages of forty-five and forty-nine, of very plethoric habits, and each of them had experienced the inconvenience which so often follows the cessation of the menses. They wheezed much, and their breathing was oppressed upon every motion of their bodies. Without taking any measure pointed out by the actual condition of their habits, and from being informed only that they had asthma, they adopted the practice of smoking Stramonium, and became rapidly worse. Pneumonic inflammation affected one, and intolerable head-aches with dimness of sight attacked the other. They however obtained relief by the active application of the necessary treatment.

An elderly man, whose complicated disorders had began with obstructed liver three years before, was icteric, and anasarcous, with a hard belly, and irregular pulse, and had not lain horizontally for several weeks. His respiration was laborious, and he could not leave his bed without much increased agitation. I had seen him once two weeks before; and I was called to him again in the present state. I found that that he had been smoking Stramonium for the last two days, and he died the night after I saw him without taking medicine.

Instances of patients in hydrothorax who had applied to the fumes of Stramonium, must have occurred very often to practitioners in this town during the last three months. I have seen six cases of this kind, and I am confident that at least half of them were so quieted by the practice, the force of the circulation through the lungs was so reduced, and the irritability of the frame so far exhausted, that they died prematurely as regarded the state of the disease.

The patients who suffer injurious or fatal consequences from smoking Stramonium, are chiefly those who have apoplectic or paralytic habits; young persons, affected with insidious spasmodic breathing, but who are actually consumptive; and elderly persons whose protracted complaints had ended in hydropic effusion in the chest. The effects of Stramonium must be referred, as Cullen has remarked, to its narcotic power; and if it be considered how universally the practice of smoking this herb has been diffused by the exertions of selfish interest, or of ignorant enthusiasm; the mischief that health and life have suffered from its use may be conceived, but cannot be very readily estimated.

I have had reported to me many deaths from smoking Stramonium, and I have verified many facts of this kind, without attending to doubtful effects in cases that might have been lost without its influence. I do not go into these cases, but have spoken only of what I have seen.

I am, dear Sir,
With great esteem,
Your most faithful
R. Bree.

---

Dr. Gooch, of Croydon, has kindly communicated the following cases, in three of which smoking the Stramonium appears to have effected present relief.

Mr. L. 22 years old, for the last four years has had a violent difficulty of breathing, attended by wheezing and cough, which attack him suddenly when in bed, or at meals, disables him from business, and sometimes continues more than a week. It occasionally seizes him so violently that he is unable to speak, and appears to be threatened with instant suffocation. He has had much medical advice without receiving material benefit. He now smoked the thorn-apple, swallowing the saliva and smoke; by these means the fit terminates in a few minutes. He smokes every day, even when the fit does not not occur. Sometimes it attacks him whilst dining in company, in which case, he retires, smokes a pipe-full, and returns to his friends breathing freely.

Mr. I. a short, fat, puffy man, about thirty-six years old, has been subject to a difficulty of breathing for twenty years. It comes on suddenly, sometimes after any strong exercise, and sometimes whilst in bed, continues several hours or days, and not unfrequently with a degree of severity that disables him for business. He has used various remedies by the advice of various practitioners, with little or no relief. For the last few months he has smoked the stem of the Thorn-apple, which generally removes the difficulty of breathing within half an hour, without producing giddiness or any other unpleasant effect.

A young gentleman, about fifteen years old, came to my house to day, Sunday, March 3, breathing with great difficulty. He has been subject to asthma as long as he can remember, and formerly scarcely passed a month without having a fit, which lasted him from two or three days to a week. For the last two years he has been free from the complaint, until about a fortnight ago, when he had a fit which continued about a day. Half an hour ago he was seized with another, which was severe. I made him sit down and smoke a pipe full of Stramonium; he soon began to breath with greater freedom, and in about half an hour walked home quite well. He had never in his life been relieved so soon.

The complaint recurred in about a month with its former violence, and was again removed by smoking the Stramonium.

A poor boy, about fifteen years old, has been subject to asthma for the last eight years. He is seldom free from it longer than a fortnight. It almost always attacks him in the night, waking him with cough and wheezing, which generally last four or five days. It is sometimes produced by going into a barn, from the fine dust which is raised by threshing corn. When I first saw him he was wheezing and breathing with much difficulty. The next morning he procured some Stramonium, smoked a pipe full, and remained free from complaint for several days. His mother was doubtful whether the relief was to be attributed to the remedy, as the difficulty of breathing was diminishing before it was used.

A few days afterwards, he awoke about four o'clock in the morning with violent cough and wheezing. He said that "his father got up, struck a light, and brought up stairs a pipe full of the herb; he sat up in bed and smoked it. As the spittle and smoke went down it cleared his stomach, and he laid down and slept quietly till seven o'clock, when he awoke quite well." Smoking always makes him giddy.

I saw

I saw him about a week afterwards; he had had another attack this morning and smoked a pipe full, but with less relief than before. In the evening of the same day I met him accidentally, breathing with much labour. He tried a pipe when he arrived home, but without any benefit. He has tried it several times since without any relief.

Comparing this evidence of impartial medical characters, interested in upholding the dignity of their profession, and zealous in extending its utility, with the statements of cases by patients, and individuals only *commercially* interested in the sale of the remedy, we cannot hesitate in deciding against the practice of smoking Stramonium in the more severe and urgent forms of asthma, and phthisical complaints. It is not attempted to deny that relief has in some instances followed its use, but the preceding facts prove that relief to be trifling indeed, when balanced with the mischief which has been effected. Ranking the herb, as we must do, amongst the narcotic poisons, we might, *a priori*, suppose that its essential qualities being copiously applied in the diffusible form of smoke to a very large surface minutely supplied with nerves, the paroxysms of a convulsive cough might be quieted, but at the same time, fatal injury might be induced on the sensorium. That this is the case is fearfully demonstrated by the somnolency, epilepsy, mania, and apoplexy, which have been evidently occasioned by the remedy. Again, it is proved by experiments that respiration is influenced by the brain, and ceases altogether when the functions of that organ are destroyed. Now if Stramonium does not always disturb or destroy these vital and essential functions, it is only when it is not applied in sufficient quantity, when the quality is impaired, or from some peculiar idiosyncracy of the patient.

The limits of this paper necessarily forbid a minute inquiry into the various causes of asthma; but the most narrow experience suffices to inform us, that unless these are removed, the disease will recur, however its symptoms may for a time be palliated. Asthma frequently depends upon effusion of serum or mucus in the cavities of the chest, and of the pericardium, in the bronchial tubes and air cells; upon the mal-formation of the chest; upon a diseased state of lungs; upon plethora occasioning pressure and thus impeding respiration; upon extraneous substances interrupting the natural action of the lungs, &c. all of which are most clearly and scientifically investigated and described in Dr. Bree's well known treatise on the subject. When any of these causes operate, can we rationally hope to obtain relief by destroying a series of actions induced in the system, to remove such noxious and offending agents?

Some people indeed are so tenacious of life that they seem to survive the effects of any sort of practice: in some habits nature is so indulgent, that they will recover although the treatment pursued is directly opposite to that which is dictated by reason or sanctioned by experience. Thus in fevers of a similar form and type, we see some patients recover. who have sustained the diffusible stimuli of John Brown; and others the large bleeding and drastic purgatives recommended by certain practitioners, even in the most advanced stage of the disorder.

Let then those worthy gentlemen who, from motives of mistaken humanity, have published their cases, and circulated their boasted cures throughout the empire, be cautious how they persevere in the practice which they so fearlessly recommend. If learned and skilful professional men are slow to admit dubious and dangerous remedies into their practice, surely those who have no pretensions to medical knowledge should be on their guard, not to deceive themselves, by imagining that because they have escaped with impunity, they may at all times be so favoured, or that their friends may be equally fortunate.

